# Assessment of Use and Preferences Regarding Internet-Based Health Care Delivery: Cross-Sectional Questionnaire Study

**DOI:** 10.2196/12416

**Published:** 2019-05-16

**Authors:** Georgios Paslakis, Josefine Fischer-Jacobs, Lars Pape, Mario Schiffer, Raoul Gertges, Uwe Tegtbur, Tanja Zimmermann, Mariel Nöhre, Martina de Zwaan

**Affiliations:** 1 University Health Network Toronto General Hospital Toronto, ON Canada; 2 Department of Psychiatry University of Toronto Toronto, ON Canada; 3 Department of Psychosomatic Medicine and Psychotherapy Hannover Medical School Hannover Germany; 4 Department of Pediatric, Liver and Metabolic Diseases Hannover Medical School Hannover Germany; 5 Project Kidney Transplantation 360° Hannover Medical School Hannover Germany; 6 Department of Nephrology and Hypertension Hannover Medical School Hannover Germany; 7 Department of Nephrology University Hospital Friedrich-Alexander University Erlangen Germany; 8 Department of Sports Medicine Hannover Medical School Hannover Germany

**Keywords:** telemedicine, health care delivery, internet, eHealth, teleconference, electronic medical records, online psychotherapy

## Abstract

**Background:**

There has been an incremental increase in the use of technology in health care delivery. Feasibility, acceptability, and efficacy of interventions based on internet technologies are supported by a growing body of evidence.

**Objective:**

The aim of this study was to investigate use and preferences in the general adult population in Germany for remote, internet-based interaction (eg, email, videoconferencing, electronic medical records, apps).

**Methods:**

A nationwide cross-sectional questionnaire survey in adults that was representative in terms of age, sex and educational level was carried out.

**Results:**

A total of 22.16% (538/2428) of survey participants reported not using the internet for work or private use. The nonuser phenotype can be described as being older, having lower educational and income status, and living in less populated areas. The majority of participants within the cohort of internet users reported that they would not consider using electronic medical records (973/1849, 52.62%), apps (988/1854, 53.29%), or emails to report symptoms (1040/1838, 56.58%); teleconference with one (1185/1852, 63.98%) or more experts (1239/1853, 66.86%); or participate in video psychotherapy (1476/1853, 79.65%) for the purpose of medical consultation or treatment. Older age and lower educational level were the most robust predictors of assumed future denial of use.

**Conclusions:**

Our results point toward low use and preference rates among the general population for the use of telemedicine. It also seems that those who might benefit from telemedical interventions the most, are, in fact, those who are most hesitating. These low use and preference rates of eHealth should be considered prior to designing and providing future telemedical care, supporting the need for easy-to-use, data secure solutions.

## Introduction

### Background

With technological innovations permeating all aspects of life, there has been an incremental increase in the use of technology in health care delivery. Feasibility, acceptability, and efficacy in terms of symptom improvement or improvements in quality of life using interventions based on internet technologies are supported by a growing body of evidence [1-4]. Patient access to online tools implemented to address chronic conditions (diabetes mellitus, asthma, etc) has improved patient self-care [5-7], with a main advantage being self-care delivery in low- and middle-income countries [8-10]. In order to best exploit the advantages while simultaneously avoiding the pitfalls, the US government has issued a mandate for the appropriate use of health care technology [11].

Beside the implementation of internet-based interventions used by patients on their own independent of any patient-physician interaction, the actual (mostly real time, depending on definition) practice of medicine through the internet involving a physician-patient interaction, known as telemedicine, presents an opportunity to revolutionize health care delivery on a global scale [12]. While early work has focused on the delivery of telemedicine services on an organizational level (eg, clinic-to-clinic teleneurology or telestroke [13]), more recently telemedicine has expanded to the delivery of health care services directly to a patient’s home. Frequently called direct-to-consumer care, this kind of telemedicine, in which a patient interacts with a physician or another health care specialist via email or videoconferencing, for example, is growing rapidly [14,15].

In many countries, direct-to-consumer telehealth companies offer patients with minor illnesses around-the-clock access to a physician. In Germany, health insurance companies offer telephone or guided internet support for specific illnesses such as depression or tinnitus, while an increasing financial grant support is dedicated to the investigation of the potential benefits of different types of telemedicine services [16]. In other European countries, for example Estonia, electronic medical records have already been implemented. Patient-accessible electronic health records are thought to increase patient involvement in their own health care matters as access to information increases [17]. Still, direct-to-consumer telemedicine requires patients to use potentially unfamiliar technology, and data security aspects may restrict its use in view of privacy and liability concerns. It is therefore important to examine the perspective of the population toward telemedicine prior to the implementation of definite solutions. On the provider side, costs for infrastructure and the mobilization of resources (eg, need for trainings) are some of the potential limiting factors for the implementation of telemedicine services. The economic advantages of such internet-based services remain unclear; also, even though remote consultation or treatment may be less expensive than personal visits, new use may not decrease overall health care spending [18].

### Need for the Study

In order to facilitate a safe, timely, efficient, effective, and equitable delivery of internet-based health care, assessment of needs of all stakeholders involved is crucial [19]. Most studies so far have intended to shed light on the provider point of view, mostly on an institutional level, in developed and developing countries ([20], systematic review in [21]), while the recipient point of view has been scarcely examined, mostly in patient cohorts with very specific needs living with chronic conditions [22,23]. Early efforts to implement the electronic health card and corresponding telematic infrastructure are in progress in Germany, and some health insurance companies have established electronic medical records, but German use and preferences regarding internet-based health care delivery has not been assessed. Therefore, in a hypothesis-free manner, the aim of this study was to investigate use and preferences of the general adult population in Germany by means of a questionnaire about remote, internet-based interaction (email, videoconferencing, electronic medical record, apps) with medical professionals. The goal was to gather data and stratify them according to sociodemographic variables and in the light of benefit-related aspects, thus serving as a basis for the planning and implementation of future telemedical solutions.

## Methods

### Recruitment

Participants in the study were part of a larger cross-sectional survey on physical and mental well-being in a random sample of German residents aged 14 years and older (range 14 to 91 years). The demographic consulting company USUMA (Unabhängiger Service für Umfragen, Methoden und Analysen) assisted with sampling and data collection. The procedure was designed to yield a nationwide sample representative in terms of age, sex, and educational level over the fieldwork period from May to July 2018. Sociodemographic data were collected in person by trained interviewers. In addition, participants returned a battery of self-report questionnaires including the telemedicine questions. This study was part of a larger survey assessing different issues in the general population for research purposes in Germany. For the purposes of this study, we assessed adults only. Thus, only participants aged 18 years and older were included in the analyses.

### Data Acquisition

In Germany, no directory is generally available containing the addresses of all private households or individuals that can be used by market research agencies as a sampling frame. The data collected by the local authorities are only available for surveys considered to be of major public interest.

The consortium Arbeitsgemeinschaft ADM-Stichproben closes this gap by providing a sampling frame, the ADM Sampling System for Face-to-Face Surveys, to member agencies. The demographic consulting company, USUMA, supporting this study is a member agency and has access to this sampling system [24]. This frame allows representative face-to-face samples to be drawn for all households in Germany and for all people living in those households. The main statistical data are provided on a detailed level for this population.

The ADM Sampling System is organized as an area sample comprising all populated areas in Germany, organized by state, county, and community with the statistical areas within communities described by public data and the geographical data taken from traffic navigation systems. Taken together, the area sample consists of about 53,000 areas, each containing a minimum of 350 and an average of 700 private households. All areas were first regionally stratified resulting in approximately 1500 strata. Next, 128 nonoverlapping nets were randomly extracted containing a total of 258 areas across Germany. These 258 areas were drawn proportionally to the distribution of private households. Since the sampling is done randomly in three steps (first step: stratified drawing of a sample point system after random allocation, second step: random-walk household selection procedure, third step: Kish-Selection-Grid method for randomly selecting the target person within the household), this method for face-to-face surveys is based entirely on random sampling and fully meets the scientific requirements regarding randomization based on statistical theory [24].

The participation rate was 47.3%, taking into account all refusals to participate as well as interviews that failed to take place due to respondent illness or being otherwise unavailable during the fieldwork. All participants provided their written informed consent in accordance with the Helsinki declaration. The study was approved by the ethics committee of the University of Leipzig.

The following sociodemographic data were assessed: Sex (male and female), age (distinguished according to groups: 18-24, 25-34, 35-44, 45-54, 55-65, >65 years), educational level (<12 and ≥12 years of education), monthly income (0 to <1000, 1000-2500, and ≥2500 euros/month), population size (<5000, 5000-50,000, and ≥50,000 residents).

Participants were first asked about their internet use, email use, and use of videoconference technologies (eg, Skype) in general. Participants were asked to choose between never, rarely (sporadic), frequently (on single days of the week), regularly (on most days of the weeks), and daily (every day) for internet use and yes or no for email and videoconference use.

Participants who reported at least sporadic use of the internet (internet users) were then asked a series of 7 pairs of questions about the medical context of consultation or treatment. Questions were constructed and chosen from a larger pool by physicians and psychologists at the Department of Psychosomatic Medicine and Psychotherapy at Hannover Medical School who have previously been involved in internet-based studies [16,25-27]. Affirmative (yes) answers about the use of internet-based health care delivery were considered to reflect preferences, while no answers to the same questions were considered indicative of reluctance. All questions were short, target-oriented, and simple:

1. Would you use/have you used email to schedule visits with your physician?
2. Would you use/have you used email to report symptoms to your physician?
3. Would you use/have you used videoconferencing with your physician?
4. Would you use/have you used videoconferencing with more than one physician (eg, general practitioner and specialist) at the same time?
5. Would you use/have you used videoconferencing for psychotherapy?
6. Would you use/have you used electronic medical records you can access at any time to see your exam results and leave messages?
7. Would you use/have you used an app that offers personalized information about your condition and recommends exercises and support?

### Statistical Analyses

Statistical analyses were performed using SPSS Statistics for Windows version 25.0 (IBM Corp). Analyses of variance or *t* tests for comparisons between groups were performed appropriately with sociodemographic data as between-subject factors or independent variables, respectively. In order to examine the predictive value of the independent variables, binary logistic regressions and multiple linear regressions were performed for dependent variables consisting of two or more than two categories, respectively. The level of significance was set at *P* ≤.05, but a Bonferroni correction for multiple testing was performed according to the number of independent variables in each hypothesis testing.

## Results

### Cohort

A total of 2516 individuals participated in the study. Of those, 77 were excluded for being younger than 18 years. Thus, data from a total of 2439 adults were analyzed. This cohort consisted of 45.10% (1100/2439) males and 54.90% (1339/2439) females. Mean age was 49.04 (SD 16.87) years, and 40.01% (976/2439) of participants were aged older than 55 years. Almost 4 out of 5 participants (1926/2439, 78.97%) had less than 12 years of education, with 27.71% (662/2439) belonging to a low-income group and the majority (1487/2439, 62.24%) having an average income of 1000 to <2500 euros per month. More details on sociodemographics of the cohort are shown in Table 1.

**Table 1:** Sociodemographics of study participants as a cohort.

Variables		Survey participants, n (%)
**Sex (n=2439)**		
	Male	1100 (45.10)
	Female	1339 (54.90)
**Age in years (n=2439)**		
	18-24	196 (8.04)
	25-34	372 (15.25)
	35-44	423 (17.34)
	45-54	472 (19.36)
	55-64	483 (19.80)
	65+	493 (20.21)
**Education, years (n=2439)**		
	<12	1926 (78.97)
	≥12	513 (21.03)
**Income (euros/month) (n=2389)**		
	0 to <1000	662 (27.71)
	1000 to <2500	1487 (62.24)
	≥2500	240 (10.05)
**Population (n=2439)**		
	<5000	351 (14.39)
	5000 to <50,000	1028 (42.15)
	≥50,000	1060 (43.46)

### Internet, Email, and Videoconference Use in General

When asked if they used the internet in general for work or for private purposes, 2428 responded (11 participants with missing data). Of these, 22.16% (538/2428) denied using the internet (nonusers), while 77.84% (1890/2428) said they used the internet for work or in private (internet users); 43.2% use the internet on a daily basis.

**Figure 1:**
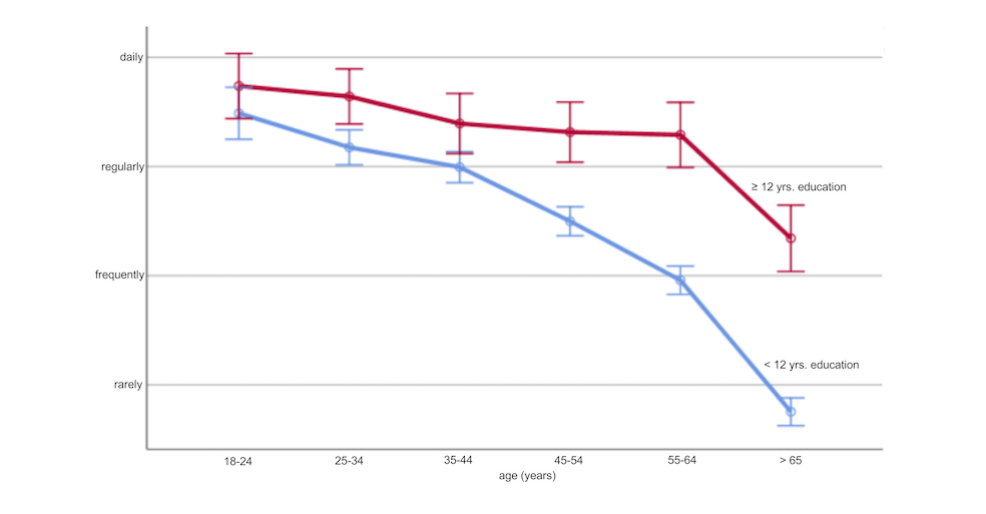
Internet use depending on age and educational level in a survey of adults from the general population. A significant interaction between age and educational level is found.

Among the 2428 respondents, there was no difference in the frequency of internet use in general by sex (*P*=.30). However, age group revealed a significant main effect (F_5,2422_=189.55, *P*<.001), with linearly declining internet use frequency parallel to increasing age. Similarly, a significant main effect was found for educational level (*P*<.001), with lower rates of internet use frequency in individuals with less than 12 years compared to those with 12 years or more of education. In fact, there was a significant interaction between age group and educational level (F_5,2416_=10.32, *P*<.001) (Figure 1).

Higher income corresponded to higher internet use frequency (F_2,2375_=25.33, *P*<.001; Games-Howell post hoc: highest income group > middle-income group = low-income group) (data not shown). Finally, there was also a significant main effect for population size (F_2,2425_=11.80, *P*<.001; Games-Howell post hoc: small communities/cities < middle size cities < big cities), with higher rates of internet use in larger cities (data not shown).

In a multiple linear regression to predict internet use frequency based on sex, age, educational level, income, and population size, a significant regression equation was found (F_5,2372_=213.71, *P*<.001), with an *R*^2^ of .31. Age, educational level, income (all *P*<.001), and population size (*P*=.001) were significant predictors of internet use frequency and remained significant even after Bonferroni correction with the new level of significance set at .05/5=.01. However, sex was not a significant predictor of internet use frequency (Table 2).

**Table 2:** Linear regression analysis to predict internet use based on sex, age, income, population size, and educational level.

Variable	Nonstandardized coefficient		Standardized coefficient beta	*t*-value	*P* value
	Regression coefficient B (SE^a^)	95% CI			
Constant	4.09 (.18)	3.74 to 4.44		22.81	<.001
Sex	.11 (.06)	–0.002 to 0.23	.03	1.93	.054
Age	–.50 (.02)	–0.54 to –0.47	–.49	–28.17	<.001
Education	.63 (.07)	0.49 to 0.77	.16	8.94	<.001
Income	.39 (.05)	0.29 to 0.49	.14	7.66	<.001
Population	.14 (.04)	0.06 to 0.22	.06	3.45	.001

^a^SE: standard error.

### Internet Users

Comparing internet users and nonusers, no differences in sex distribution or population size of the community or city of origin were found. On the other hand, age distribution, educational level, and income differed (eg, more than half of nonusers were aged 65 years and older, most of them had an educational level of less than 12 years, and there more nonusers belonging to the low-income group compared to internet users). More details are shown in Table 3.

Within the group of internet users, 6.85% (129/1884, 6 missing) stated they did not use email, while 93.15% (1755/1884) did. Within the same group of internet users, the majority (58.71%, 1089/1855, 35 missing) did not use videoconference programs, and 41.29% (766/1855) were familiar with their use.

### Internet Users Versus Nonusers

**Figure 2:**
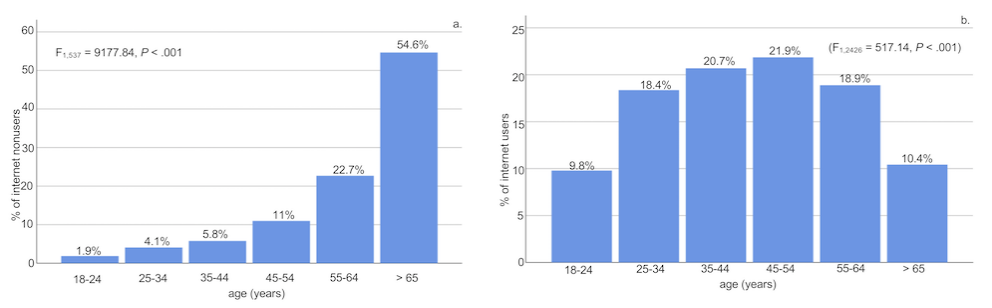
Distribution of age groups in noninternet users (a) compared to internet users (b). Within each of the groups users versus nonusers, age groups had a significant main effect. Age groups also differed between the groups.

**Figure 3:**
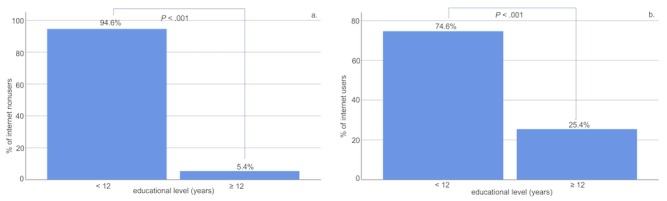
Comparisons of educational levels in noninternet users (a) compared to internet users (b). Within each of the groups users versus nonusers, educational level was significantly different with regard to the total years of education. Educational level also differed between the groups.

Within the group of internet users, age had a significant main effect (F_1,1889_=10,664.20, *P*<.001) and educational level was significantly different with regard to the total years of education (*P*<.001). Similarly, age had a significant main effect (F_1,537_=9177.84, *P*<.001) and educational level was significantly different with regard to the total years of education (*P*<.001) within the group of nonusers. These results are displayed in Figures 2 and 3.

**Table 3:** Sociodemographic characteristics of internet users and nonusers.

Variables		Internet users, n (%)	Internet nonusers, n (%)	df^a^	Chi-square	*P* value
**Sex (n=1890)**				**1**	**0.6**	**.44**
	Male	861 (45.56)	235 (43.68)			
	Female	1029 (54.44)	303 (56.32)			
**Age in years (n=1890)**				**5**	**576.17**	**<.001**
	18-24	185 (9.79)	10 (1.86)			
	25-34	347 (18.36)	22 (4.09)			
	35-44	391 (20.69)	31 (5.76)			
	45-54	413 (21.85)	59 (10.97)			
	55-64	357 (18.89)	122 (22.67)			
	65+	197 (10.42)	294 (54.65)			
**Education in years (n=1890)**				**1**	**101.17**	**<.001**
	<12	1410 (74.60)	509 (94.61)			
	≥12	480 (25.40)	29 (5.39)			
**Income in euros per month (n=1845)**				**2**	**28.9**	**<.001**
	0 to <1000	486 (26.34)	169 (31.70)			
	1000 to <2500	1141 (61.84)	342 (64.17)			
	≥2500	218 (11.82)	22 (4.13)			
**Population (n=1890)**				**2**	**3.33**	**.19**
	<5000	261 (13.81)	88 (16.35)			
	5000 to <50,000	790 (41.80)	231 (42.94)			
	≥50,000	839 (44.39)	219 (40.71)			

^a^df: degree of freedom.

### Telemedicine Within the Medical Consultation and Treatment Context

The 1890 internet users were asked whether they would consider using email, videoconferencing, electronic patient charts, or apps within a medical context of consultation or treatment in the future and whether they have already made use of any of these options.

The majority of participants answered that they would take advantage of using email for scheduling medical visits (1257/1890, 66.51%), although the majority of participants have not used email for this purpose so far (1247/1890, 66.00%). In contrast, most participants would not use email for reporting symptoms to their physicians (1040/1890, 55.03%), and few participants (64/1890, 3.39%) have done this in the past.

The majority of internet users also proved to be reluctant to use videoconferencing for consultations with their physician (1185/1836, 64.54%), and only 0.76% (14/1836) reported having had experience with it. Similarly, consultation with more than one physician at once by means of videoconference would not be considered by most internet users (1239/1836, 67.48%). Only 1.35% (19/1836) had actual experience with psychotherapy using videoconference technology, and most users reported low preference rates for online video-psychotherapy (377/1836, 20.53%). Electronic patient charts were not familiar to most users (1547/1836, 84.26%), but a slight majority would consider electronic patient charts as a future option (973/1836, 53.00%). Almost half of internet users would use a medical app (866/1836, 47.17%); however, just 1.85% (34/1836) have already done so.

In Table 4, more details about the results concerning the willingness of adult internet users to consider the use of internet technologies within a medical context of consultation or treatment as well as their actual experiences with technologies of this kind within this specific context are displayed.

Next, binary logistic regression analyses were performed for the use of internet technology within a medical context based on sex, age, educational level, income, population size, and previous experience with the respective online tool under investigation. Due to the insertion of 6 independent variables in the regression equation, the new level of significance was set at .05/6=.008.

Regarding email use for scheduling future medical visits, a significant regression model was found, χ^2^_12_=296.4, *P*<.001. Age, educational level, and previous experience with email for this purpose were significant predictors (*P*<.001) as was population size (*P*=.007). Sex and income (following Bonferroni correction) were not significant predictors.

For using email for reporting symptoms, the logistic regression model turned out to be significant, χ^2^_12_=137.5, *P*<.001. Age, educational level, and previous email use for reporting symptoms were significant predictors (all *P*<.001). Sex, income, and population size were not significant predictors at the new level of statistical significance.

For videoconference use to communicate with a physician, the regression model was significant, χ^2^_12_=62.8, *P*<.001. Age (*P*=.03), educational level (*P*<.001), and income (*P*=.005) were significant predictors whereas sex, population size, and previous experience with teleconference were not.

The regression equation for the possibility of videoconference use with more than one physician at once turned out to be significant χ^2^_12_=77.0, *P*<.001. Age (*P*=.001), educational level (*P*<.001), and previous experience with group teleconference in a medical context (*P*=.008) were significant predictors. Sex, income and population size were not significant predictors.

The regression model for prediction of psychotherapy to be considered by means of videoconference also proved to be significant χ^2^_12_=60.1, *P*<.001. Here, only educational level (*P*=.002) and previous experience with online video psychotherapy (*P*<.001) were significant predictors.

Preferences regarding the use of electronic patient files were statistically predicted by age (*P*=.001), educational level (*P*<.001) as well as previous use of electronic patient files (*P*=.006) in a significant regression equation χ^2^_12_=77.0, *P*<.001.

**Table 4:** Willingness of adult internet users (n=1836) to consider the use of internet technologies within a medical context of consultation or treatment and their actual experiences with technologies of this kind within this specific context.

Variable	Yes, n (%)	No, n (%)
Would use email to schedule visits	1257 (68.46)	579 (31.54)
Have used email to schedule visits	358 (22.31)	1247 (77.69)
Would use email to report symptoms	798 (43.42)	1040 (56.58)
Have used email to report symptoms	64 (4.04)	1519 (95.96)
Would use videoconferencing with their physician	667 (36.02)	1185 (63.98)
Have used videoconferencing with their physician	14 (0.89)	1561 (99.11)
Would use videoconferencing with more than 1 physician at the same time	614 (33.14)	1239 (66.86)
Have used videoconferencing with more than 1 physician at the same time	16 (1.02)	1559 (98.98)
Would use videoconferencing for psychotherapy	377 (20.35)	1476 (79.65)
Have used videoconferencing for psychotherapy	19 (1.21)	1554 (98.79)
Would use electronic medical records	876 (47.38)	973 (52.62)
Have used electronic medical records	22 (1.40)	1547 (98.60)
Would use apps	866 (46.71)	988 (53.29)
Have used apps	34 (2.20)	1508 (97.80)

Finally, considering app use within a medical context was once more statistically predicted by age and educational level (both *P*<.001) as well as previous app experience (*P*=.001) in a significant regression model χ^2^_12_=90.5, *P*<.001.

In all models, younger age, higher educational level, higher income, and previous experience with the internet-based interventions in question were associated with higher rates of preferences for future use of internet-based solutions. All regression models can be found in Multimedia Appendix 1.

## Discussion

Internet-based solutions to provide health care consultation or even treatments have been shown to improve patient activation and engagement [28-32] and thus improve outcomes [33-35]. However, as most scientific studies have examined clinical cohorts with very specific needs (eg, patients suffering from chronic diseases), data on use and future preferences in the general population regarding internet-based solutions are scarce [21,36]. Obviously, personal preferences may differ depending on the actual individual need for medical consultation or treatment. The assessment of personal preferences and especially stratification according to specific variables may be important prior to designing and providing internet-based medical consultation or treatment approaches, the purpose being a consistent or repeated rather than a singular or just transient use.

### Principal Findings

Even in a country as technologically developed as Germany, it was quite a surprising finding that 22.5% of survey participants reported they did not use the internet for work or private use. In contrast, in a recent survey conducted in 2017 by the Federal Statistical Office (Destatis) in Germany, internet use was above 90% in all age groups except age 65 and older, in which the respective percentage was 55% [37]. We explain this obvious discrepancy with the way the questions were asked; while the Federal Statistical Office asked about lifetime internet use, our questionnaire addressed current ongoing use. According to our results, those individuals with a low preference for the implementation of internet-based health care delivery can be described as being older and having lower educational and income status compared to those individuals who expressed a preference and were younger, more educated, and had a higher income. Since age, educational level, and living in remote (rural) areas are associated with lower utilization rates of medical services and thus with higher morbidity and mortality rates [38-40], it becomes obvious that designing and providing future internet-based telemedical solutions aimed at reaching a wide-ranging and region-wide number of recipients should take the above nonuser phenotype into serious consideration. It would therefore be necessary to develop easy-to-use solutions (eg, tablet-based) suitable for those individuals who are not familiar with technology but would still like to try following simple instructions. For those without access to either the internet or computers and tablets, further solutions are needed (eg, internet hot spots, supply of tablets to the elderly or similar).

In addition, and also rather surprisingly, the majority of participants within the subcohort of internet users reported that they would not consider the use of email, videoconference, video psychotherapy, electronic medical records, or apps for the purpose of medical consultation or treatment, with the exception of using emails to schedule medical visits. This finding may point toward the fact that internet use has not been associated with the idea of a tool helping to address medical issues and needs within the same context by the general population—not even by its very own regular consumers. There is a mismatch between everyday use of internet-based technologies (email, apps, skype, etc) and willingness to use the exact same technologies for the sake of delivering health care. This is important to consider prior to implementing internet-based health care delivery solutions in Germany. Many individuals may not be able to anticipate how such systems would function with success. Data security may also be an issue; a recent market research study in Germany revealed that 95.2% of all respondents indicated fearing a possibly fraudulent use of their personal data on the internet [41]. With older age and lower educational level in our study being the most robust predictors of assumed future use, it seems as those who are supposed to benefit from telemedical interventions the most, are, in fact, those who might not use it. Thus, the development of simple, secure systems as described above might be able to overcome these obstacles.

This is a study examining use and preferences regarding a variety of internet-based technologies for health care delivery in a large representative sample in Germany. A limited number of similar studies have been performed in convenience samples, with the associated risks of sampling error and lack of representativeness inherent in convenience samples. Apolinário-Hagen et al [42] performed a Web-based study in a convenience sample (N=646) examining general preferences with reference to internet-based therapy and found similar rates of interesting in psychotherapy by means of videoconference (22.8% compared to 20.3% in our cohort). Interestingly, attachment avoidance and stress were associated with preference to internet-based interventions, and those individuals who were well aware of internet-based approaches showed a higher preference for therapist-guided internet treatment. The authors highlight the importance of increasing public knowledge about internet interventions in order to promote acceptance and uptake [42]. In another convenience sample in German and Austrian citizens (N=496), Hoerbst et al [43] examined knowledge, expectations, fears, and barriers toward electronic health records. In contrast to our findings of 47.4% of respondents being open to the idea of electronic medical records, they found more than 80% of respondents being supportive of the idea, although data protection issues were the major concerns. Our results showing low interest rates among the general population for internet-based technologies for telemedical purposes are in accordance with the results by Jenssen et al [44], in a US national survey, showing that despite regular use of new digital technology, few participants would consider the use of these tools for communicating with their physicians. In another US nationwide survey assessing participant preferences for telemedicine, Welch et al [45] found that 41% percent of survey participants felt it to be unimportant if their current health care professional offered telemedicine, only 15% would consider changing to a new professional who would provide telemedical care, and 56% of participants felt it was important to have an established relationship with a physician before considering telemedical visits. White race and higher education and relatively younger age and higher income have been described as predictors for internet use and eHealth literacy among older adults (aged 55 years and older) who were patients at clinics serving low-income populations [46]; policy makers are asked to consider such findings prior to the implementation of eHealth programs in order to meet the needs of people living different realities.

In our study, a significantly higher proportion of the population reported interest in using new telemedical technologies compared to the small minority that reported actual previous experience. Prior use was dependent on whether such technology-based solutions were provided by their practitioners and whether the need for such a use had actually existed in the past. This fact could be interpreted as insufficient exposure to the respective technologies so far; in other words, specific offers might increase specific demand. If recipients of telemedical care solutions were to experience short-term advantages of such systems (eg, being able to schedule appointments online at any time of the day, being able to see examination results and have easy access to further personal medical data) in an uncomplicated manner combined with maximum security of the systems, higher levels of actual use would be expected. Countries such as Estonia have shown that user-friendly internet-based health solutions are accepted by the majority of the population. It may therefore be expected that the implementation of simple-to-use, data-secure systems in Germany might lead to much higher use rates than shown here.

Another survey in Germany investigated preferences for online interventions in psychiatry and psychotherapy between health care professionals and nonprofessionals and found that, overall, nonprofessionals were more skeptical in their ratings than professionals [47]. More favorable eHealth utilization rates by European general practitioners were associated with younger age of the practitioners, female sex, and specific working conditions (eg, self-employment) in the study by Torrent-Sellens et al [48]. Although assessment of preferences of the medical staff involved was not part of our survey, aspects such as changes in roles and responsibilities and need for additional resources, reimbursement, and training may be crucial for successful implementation of new health care delivery approaches [49] and should be targets of investigation in further studies.

Blended treatments—the use of online tools (eg, self-help) combined with face-to-face psychotherapy—are already part of clinical routines aimed at increasing the impact of psychotherapy [50]. Nevertheless, computerized treatments and mobile phone apps for mental health problems seem to be negatively viewed and the likelihood of their future use to be rated low, indicating that policy makers need to improve the public perception of such options in order to facilitate their dissemination [51]. In a recent study in Austrian psychotherapists, both recipients of internet-based psychotherapy and their providers associated the new modes of treatment with more disadvantages and risks [52]. Again, assessment of barriers to and facilitators of use for remote psychotherapy is necessary to develop tailored, feasible, and acceptable practice designs for all stakeholders involved.

### Strengths and Limitations

Strengths of this study clearly include the large number of participants and representativeness of the cohort. However, our survey also has limitations. First, we asked the general population to express their opinions on the use of email, videoconferencing, apps, and electronic patient files, all of which are internet-based instruments intended to deliver health care. The assessment of differences between these instruments was not part of our protocol, which aimed at assessing global preferences for a variety of new technologies that may provide future meaningful contributions to improve health care delivery in the general population. In addition, the reasons for or against considering the use of internet-based telemedicine interventions were not assessed (eg, fear of dataveillance, needs remaining unmet) and may only be assumed. Other predictors should also be investigated in future studies (eg, ethnic/cultural background, strength of the physician-patient relationship); in addition, reasearch has shown that a health-related information seeking personality was a significant positive predictor of the willingness to undergo online treatment [53]. Furthermore, we did not use a validated questionnaire but a series of questions generated by experts in psychosomatic medicine. Presumably, although not explicitly assessed, a minority of the participants has been offered the use of the technologies under investigation within a medical context, as such systems are not yet fully developed or implemented in Germany in routine practice.

### Conclusion

Among internet users, the majority reported that they would not consider using internet technology for the purposes of medical consultation or treatment. Internet-based health care approaches using the right tools aimed at the right recipients may prove to be of great benefit. Our results emphasize the importance of developing solutions able to convince the general population to make proper use of and benefit from the potential telemedicine has to offer. As a putative practicable future way to increase acceptance and promote implementation of internet-based approaches in routine care, it is crucial to provide continuous, large-scale information on newer technological possibilities in health care delivery and ensure that concerns (eg, data security) are adequately addressed. In addition, policy makers should be focus on outreach to the elderly and those with lower income and educational level, as it is those groups who are more likely to report lower use of and preferences for internet-based health care delivery. Such an approach includes easy-to-use solutions and facilitation of access to internet-based services.

## Appendix

Multimedia Appendix 1 Logistic regression analyses for the prediction of attitudes toward internet-based health care delivery based on 6 predictors: sex, age, educational level, income, population size, and previous experience.
