# Supplementary material for: Assessment of Use and Preferences Regarding Internet-Based Health Care Delivery: Cross-Sectional Questionnaire Study
Source: J Med Internet Res. 2019 May 16;21(5):e12416. doi: 10.2196/12416 (PMC6542248; doi:10.2196/12416)
Supplement: Multimedia Appendix 1 [file jmir_v21i5e12416_app1.docx]

**Supplementary material: Logistic regression analyses for the prediction of attitudes towards internet-based health care delivery based on 6 predictors: sex. age group. educational level. income group. population size and previous experience (for details please refer to main text)**

**Use email to schedule visits:**

|  | | | | | | | | | |
| --- | --- | --- | --- | --- | --- | --- | --- | --- | --- |
|  | | Regression coefficient B | Standard error | Wald | df | p | Exp(B) | 95% confidence interval for EXP(B) | |
|  |  |  |  |  |  |  |  | Lower value | Upper value |
| step 1 | age groups (RG: 65+) |  |  | 22.507 | 5 | .000 |  |  |  |
|  | 18-24 | .934 | .277 | 11.414 | 1 | .001 | 2.546 | 1.480 | 4.378 |
|  | 25-34 | .745 | .226 | 10.853 | 1 | .001 | 2.107 | 1.353 | 3.284 |
|  | 35-44 | .316 | .223 | 2.017 | 1 | .156 | 1.372 | .887 | 2.122 |
|  | 45-54 | .158 | .217 | .531 | 1 | .466 | 1.171 | .766 | 1.791 |
|  | 55-64 | .148 | .218 | .464 | 1 | .496 | 1.160 | .757 | 1.778 |
|  | Educational level (RG: ≥ 12 years) | -.638 | .157 | 16.532 | 1 | .000 | .528 | .389 | .719 |
|  | Income groups (RG: ≥ 2500) |  |  | 9.391 | 2 | .009 |  |  |  |
|  | 0 to < 1000 | -.796 | .260 | 9.386 | 1 | .002 | .451 | .271 | .751 |
|  | 1000 to < 2500 | -.600 | .232 | 6.660 | 1 | .010 | .549 | .348 | .866 |
|  | Population size (RG: ≥ 50000) |  |  | 9.851 | 2 | .007 |  |  |  |
|  | < 5000 | .508 | .189 | 7.197 | 1 | .007 | 1.662 | 1.147 | 2.408 |
|  | 5000 to < 50000 | .322 | .131 | 5.988 | 1 | .014 | 1.380 | 1.066 | 1.785 |
|  | Sex (RG: females) | -.030 | .128 | .056 | 1 | .814 | .970 | .755 | 1.247 |
|  | Previous experience (RG: no) | 4.160 | .585 | 50.637 | 1 | .000 | 64.098 | 20.379 | 201.605 |
|  | constant | 1.056 | .306 | 11.948 | 1 | .001 | 2.876 |  |  |

**RG: reference group. Predicted probability is of membership for preference towards the question asked.**

**Use email to report symptoms:**

|  | | | | | | | | | |
| --- | --- | --- | --- | --- | --- | --- | --- | --- | --- |
|  | | Regression coefficient B | Standard error | Wald | df | p | Exp(B) | 95% confidence interval for EXP(B) | |
|  |  |  |  |  |  |  |  | Lower value | Upper value |
| step 1 | age groups (RG: 65+) |  |  | 28.662 | 5 | .000 |  |  |  |
|  | 18-24 | .821 | .249 | 10.915 | 1 | .001 | 2.273 | 1.397 | 3.700 |
|  | 25-34 | .921 | .215 | 18.286 | 1 | .000 | 2.512 | 1.647 | 3.831 |
|  | 35-44 | .435 | .217 | 4.039 | 1 | .044 | 1.545 | 1.011 | 2.363 |
|  | 45-54 | .455 | .214 | 4.534 | 1 | .033 | 1.576 | 1.037 | 2.395 |
|  | 55-64 | .193 | .219 | .777 | 1 | .378 | 1.213 | .790 | 1.861 |
|  | Educational level (RG: ≥ 12 years) | -.554 | .129 | 18.487 | 1 | .000 | .575 | .447 | .740 |
|  | Income groups (RG: ≥ 2500) |  |  | 7.598 | 2 | .022 |  |  |  |
|  | 0 to < 1000 | -.527 | .213 | 6.120 | 1 | .013 | .591 | .389 | .896 |
|  | 1000 to < 2500 | -.209 | .183 | 1.300 | 1 | .254 | .811 | .566 | 1.162 |
|  | Population size (RG: ≥ 50000) |  |  | 7.324 | 2 | .026 |  |  |  |
|  | < 5000 | .438 | .168 | 6.797 | 1 | .009 | 1.549 | 1.115 | 2.153 |
|  | 5000 to < 50000 | .187 | .118 | 2.516 | 1 | .113 | 1.205 | .957 | 1.519 |
|  | Sex (RG: females) | .164 | .114 | 2.065 | 1 | .151 | 1.178 | .942 | 1.472 |
|  | Previous experience (RG: no) | 2.960 | .524 | 31.844 | 1 | .000 | 19.293 | 6.902 | 53.931 |
|  | constant | -.359 | .263 | 1.862 | 1 | .172 | .699 |  |  |

**RG: reference group. Predicted probability is of membership for preference towards the question asked.**

**Use of videoconferencing:**

|  | | | | | | | | | |
| --- | --- | --- | --- | --- | --- | --- | --- | --- | --- |
|  | | Regression coefficient B | Standard error | Wald | df | p | Exp(B) | 95% confidence interval for EXP(B) | |
|  |  |  |  |  |  |  |  | Lower value | Upper value |
| step 1 | age groups (RG: 65+) |  |  | 17.721 | 5 | .003 |  |  |  |
|  | 18-24 | .696 | .252 | 7.638 | 1 | .006 | 2.006 | 1.224 | 3.288 |
|  | 25-34 | .777 | .218 | 12.744 | 1 | .000 | 2.174 | 1.419 | 3.331 |
|  | 35-44 | .439 | .221 | 3.957 | 1 | .047 | 1.551 | 1.007 | 2.391 |
|  | 45-54 | .385 | .217 | 3.139 | 1 | .076 | 1.469 | .960 | 2.249 |
|  | 55-64 | .255 | .223 | 1.308 | 1 | .253 | 1.290 | .834 | 1.996 |
|  | Educational level (RG: ≥ 12 years) | -.612 | .128 | 23.032 | 1 | .000 | .542 | .422 | .696 |
|  | Income groups (RG: ≥ 2500) |  |  | 10.634 | 2 | .005 |  |  |  |
|  | 0 to < 1000 | -.484 | .214 | 5.136 | 1 | .023 | .616 | .406 | .937 |
|  | 1000 to < 2500 | -.038 | .182 | .044 | 1 | .835 | .963 | .674 | 1.375 |
|  | Population size (RG: ≥ 50000) |  |  | 4.180 | 2 | .124 |  |  |  |
|  | < 5000 | .332 | .168 | 3.892 | 1 | .049 | 1.394 | 1.002 | 1.938 |
|  | 5000 to < 50000 | .141 | .119 | 1.414 | 1 | .234 | 1.151 | .913 | 1.453 |
|  | Sex (RG: females) | .054 | .114 | .227 | 1 | .634 | 1.056 | .844 | 1.320 |
|  | Previous experience (RG: no) | 1.036 | .649 | 2.549 | 1 | .110 | 2.818 | .790 | 10.052 |
|  | constant | -.544 | .264 | 4.243 | 1 | .039 | .581 |  |  |

**RG: reference group. Predicted probability is of membership for preference towards the question asked.**

**Use of videoconferencing with > 1 physician:**

|  | | Regression coefficient B | Standard error | Wald | df | p | Exp(B) | 95% confidence interval for EXP(B) | |
| --- | --- | --- | --- | --- | --- | --- | --- | --- | --- |
|  |  |  |  |  |  |  |  | Lower value | Upper value |
| step 1 | age groups (RG: 65+) |  |  | 21.919 | 5 | .001 |  |  |  |
|  | 18-24 | .736 | .257 | 8.184 | 1 | .004 | 2.087 | 1.261 | 3.455 |
|  | 25-34 | .769 | .222 | 12.053 | 1 | .001 | 2.158 | 1.398 | 3.332 |
|  | 35-44 | .406 | .226 | 3.241 | 1 | .072 | 1.501 | .965 | 2.336 |
|  | 45-54 | .332 | .223 | 2.210 | 1 | .137 | 1.393 | .900 | 2.157 |
|  | 55-64 | .103 | .230 | .202 | 1 | .653 | 1.109 | .707 | 1.740 |
|  | Educational level (RG: ≥ 12 years) | -.695 | .129 | 28.828 | 1 | .000 | .499 | .387 | .643 |
|  | Income groups (RG: ≥ 2500) |  |  | 9.384 | 2 | .009 |  |  |  |
|  | 0 to < 1000 | -.395 | .220 | 3.231 | 1 | .072 | .674 | .438 | 1.036 |
|  | 1000 to < 2500 | .044 | .187 | .056 | 1 | .813 | 1.045 | .724 | 1.509 |
|  | Population size (RG: ≥ 50000) |  |  | 3.341 | 2 | .188 |  |  |  |
|  | < 5000 | .315 | .173 | 3.333 | 1 | .068 | 1.371 | .977 | 1.922 |
|  | 5000 to < 50000 | .087 | .121 | .516 | 1 | .473 | 1.091 | .860 | 1.384 |
|  | Sex (RG: females) | .022 | .117 | .036 | 1 | .849 | 1.022 | .813 | 1.285 |
|  | Previous experience (RG: no) | 1.792 | .678 | 6.984 | 1 | .008 | 6.001 | 1.589 | 22.668 |
|  | constant | -.617 | .270 | 5.228 | 1 | .022 | .540 |  |  |

**RG: reference group. Predicted probability is of membership for preference towards the question asked.**

**Use of videoconferencing for psychotherapy:**

|  | | Regression coefficient B | Standard error | Wald | df | p | Exp(B) | 95% confidence interval for EXP(B) | |
| --- | --- | --- | --- | --- | --- | --- | --- | --- | --- |
|  |  |  |  |  |  |  |  | Lower value | Upper value |
| step 1 | age groups (RG: 65+) |  |  | 14.659 | 5 | .012 |  |  |  |
|  | 18-24 | .828 | .302 | 7.497 | 1 | .006 | 2.288 | 1.265 | 4.137 |
|  | 25-34 | .703 | .267 | 6.927 | 1 | .008 | 2.020 | 1.197 | 3.411 |
|  | 35-44 | .308 | .278 | 1.228 | 1 | .268 | 1.361 | .789 | 2.349 |
|  | 45-54 | .523 | .269 | 3.787 | 1 | .052 | 1.687 | .996 | 2.858 |
|  | 55-64 | .174 | .282 | .381 | 1 | .537 | 1.190 | .685 | 2.066 |
|  | Educational level (RG: ≥ 12 years) | -.451 | .149 | 9.145 | 1 | .002 | .637 | .476 | .853 |
|  | Income groups (RG: ≥ 2500) |  |  | 4.267 | 2 | .118 |  |  |  |
|  | 0 to < 1000 | -.072 | .261 | .076 | 1 | .782 | .930 | .558 | 1.552 |
|  | 1000 to < 2500 | .243 | .225 | 1.165 | 1 | .280 | 1.275 | .820 | 1.983 |
|  | Population size (RG: ≥ 50000) |  |  | 2.138 | 2 | .343 |  |  |  |
|  | < 5000 | .252 | .201 | 1.574 | 1 | .210 | 1.286 | .868 | 1.906 |
|  | 5000 to < 50000 | .163 | .142 | 1.325 | 1 | .250 | 1.177 | .892 | 1.554 |
|  | Sex (RG: females) | .067 | .135 | .244 | 1 | .621 | 1.069 | .820 | 1.393 |
|  | Previous experience (RG: no) | 2.912 | .652 | 19.917 | 1 | .000 | 18.384 | 5.118 | 66.033 |
|  | constant | -1.780 | .329 | 29.279 | 1 | .000 | .169 |  |  |

**RG: reference group. Predicted probability is of membership for preference towards the question asked.**

**Use of electronic medical records:**

|  | | | | | | | | | |
| --- | --- | --- | --- | --- | --- | --- | --- | --- | --- |
|  | | Regression coefficient B | Standard error | Wald | df | p | Exp(B) | 95% confidence interval for EXP(B) | |
|  |  |  |  |  |  |  |  | Lower value | Upper value |
| step 1 | age groups (RG: 65+) |  |  | 19.768 | 5 | .001 |  |  |  |
|  | 18-24 | .790 | .242 | 10.673 | 1 | .001 | 2.203 | 1.372 | 3.539 |
|  | 25-34 | .857 | .209 | 16.854 | 1 | .000 | 2.357 | 1.565 | 3.550 |
|  | 35-44 | .525 | .209 | 6.311 | 1 | .012 | 1.690 | 1.122 | 2.544 |
|  | 45-54 | .526 | .206 | 6.540 | 1 | .011 | 1.691 | 1.131 | 2.530 |
|  | 55-64 | .421 | .209 | 4.035 | 1 | .045 | 1.523 | 1.010 | 2.296 |
|  | Educational level (RG: ≥ 12 years) | -.698 | .128 | 29.668 | 1 | .000 | .497 | .387 | .640 |
|  | Income groups (RG: ≥ 2500) |  |  | 9.360 | 2 | .009 |  |  |  |
|  | 0 to < 1000 | -.516 | .209 | 6.106 | 1 | .013 | .597 | .397 | .899 |
|  | 1000 to < 2500 | -.147 | .181 | .656 | 1 | .418 | .863 | .605 | 1.232 |
|  | Population size (RG: ≥ 50000) |  |  | 1.730 | 2 | .421 |  |  |  |
|  | < 5000 | .151 | .165 | .835 | 1 | .361 | 1.163 | .842 | 1.606 |
|  | 5000 to < 50000 | .137 | .114 | 1.439 | 1 | .230 | 1.147 | .917 | 1.435 |
|  | Sex (RG: females) | -.159 | .111 | 2.025 | 1 | .155 | .853 | .686 | 1.062 |
|  | Previous experience (RG: no) | 1.791 | .646 | 7.687 | 1 | .006 | 5.995 | 1.690 | 21.263 |
|  | constant | .133 | .255 | .270 | 1 | .603 | 1.142 |  |  |

**RG: reference group. Predicted probability is of membership for preference towards the question asked.**

**Use of apps:**

|  | | | | | | | | | |
| --- | --- | --- | --- | --- | --- | --- | --- | --- | --- |
|  | | Regression coefficient B | Standard error | Wald | df | p | Exp(B) | 95% confidence interval for EXP(B) | |
|  |  |  |  |  |  |  |  | Lower value | Upper value |
| step 1 | age groups (RG: 65+) |  |  | 43.237 | 5 | .000 |  |  |  |
|  | 18-24 | 1.126 | .247 | 20.849 | 1 | .000 | 3.083 | 1.901 | 4.998 |
|  | 25-34 | .688 | .208 | 10.945 | 1 | .001 | 1.989 | 1.323 | 2.989 |
|  | 35-44 | .721 | .208 | 12.057 | 1 | .001 | 2.056 | 1.369 | 3.089 |
|  | 45-54 | .503 | .204 | 6.085 | 1 | .014 | 1.653 | 1.109 | 2.465 |
|  | 55-64 | -.034 | .212 | .026 | 1 | .873 | .967 | .638 | 1.464 |
|  | Educational level (RG: ≥ 12 years) | -.542 | .129 | 17.641 | 1 | .000 | .581 | .451 | .749 |
|  | Income groups (RG: ≥ 2500) |  |  | 2.923 | 2 | .232 |  |  |  |
|  | 0 to < 1000 | -.178 | .209 | .725 | 1 | .395 | .837 | .555 | 1.261 |
|  | 1000 to < 2500 | .050 | .181 | .078 | 1 | .781 | 1.052 | .737 | 1.500 |
|  | Population size (RG: ≥ 50000) |  |  | 1.518 | 2 | .468 |  |  |  |
|  | < 5000 | .101 | .168 | .361 | 1 | .548 | 1.106 | .796 | 1.537 |
|  | 5000 to < 50000 | -.095 | .116 | .671 | 1 | .413 | .909 | .724 | 1.141 |
|  | Sex (RG: females) | -.125 | .113 | 1.223 | 1 | .269 | .882 | .707 | 1.101 |
|  | Previous experience (RG: no) | 1.871 | .550 | 11.570 | 1 | .001 | 6.495 | 2.210 | 19.090 |
|  | constant | -.132 | .252 | .273 | 1 | .601 | .877 |  |  |

**RG: reference group. Predicted probability is of membership for preference towards the question asked.**
